# Luminescent concentrators enable highly efficient and broadband photodetection

**DOI:** 10.1038/s41377-022-00819-3

**Published:** 2022-05-06

**Authors:** Wei Wang, Johnny C. Ho

**Affiliations:** 1 Department of Materials Science and Engineering, City University of Hong Kong, Hong Kong SAR, PR China; 2 State Key Laboratory of Terahertz and Millimeter Waves, City University of Hong Kong, Hong Kong SAR, PR China

**Keywords:** Optoelectronic devices and components, Photonic devices

## Abstract

With luminescent concentrators, the high quantum yield luminescence emitted by embedded chromophores, featuring a broad absorption spectrum, can be well-tuned to match the peak response of integrated photodetectors. This integration can substantially enhance the device photoresponse all the way from deep UV to near-IR.

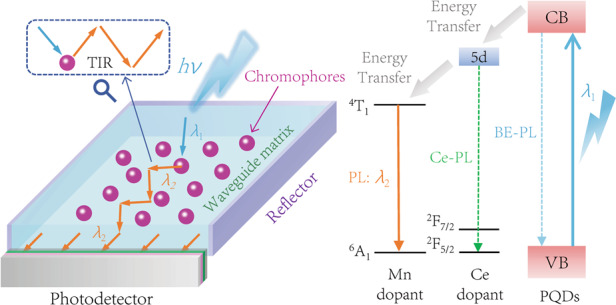

The concept of luminescent concentrators (LCs) was initially proposed in the 1970s, aiming to develop a low-cost technology of photovoltaic (PV) solar cells and enhance their spectral response at that time^1,2^. In specific, the chromophores embedded in a transparent waveguide slab can absorb the incident photons across a broad, energetic spectrum and then re-emit the downshifted light, which would then be directed to the integrated PV cells at small edges of LCs^3^. This way, the luminescent solar concentrators offer a practical path to reduce the expensive PV material consumption and enhance the spectra response range. Thanks to the unique features, such as light concentration and energy down-conversion, the LCs have also drawn considerable interest in other application fields as a versatile photonic technology platform, such as the compact dark-field imaging devices and multistate smart windows evolved by the LCs^4–6^.

Recently, significant efforts have been made to develop efficient and broadband photodetectors. Nevertheless, the capability of photo-sensing for most photodetectors has always been constrained due to the poor spectral selective response. For instance, the existing broadband photodetectors based on hybrid heterojunctions, such as PtSe_2_/GaAs or MAPbI_3_/organic PTB7-Th:F8IC, tend to exhibit prominent performance primarily ranging from visible to shortwave infrared or even only in the visible waveband, whereas the detection capability in deep UV is not so comparable and conspicuous^7,8^. Moreover, the incident diffuse light is also prone to be scattered when irradiating on the photodetectors, resulting in low external quantum efficiency (EQE).

Fortunately, with the introduction of LCs, the aforementioned drawbacks of photodetectors can be readily remedied by judicious adoption of suitable chromophores and waveguide matrices. In this case, it is possible to make more efficient use of photodetectors by integrating them with LCs. Very recently, to enhance UV performance of CsPbI_3_:Er^3+^/BTP-4Cl:PBDB-TF heterostructure photodetectors, Song’s group employed the Cr^3+^, Ce^3+^, Mn^2+^ tri-doped CsPbCl_3_ perovskite quantum dots (PQDs) as chromophores in LCs^9^. As a result of visible luminescence emission, the EQE and detectivity of LC-coupled heterostructure photodetector at 260 nm were increased by over 4 orders of magnitude, along with an efficient response time of hundreds of microseconds, successfully realizing the full spectrum of the hybrid photodetectors within 200–1000 nm.

This work demonstrates that the efficient photoresponse of photodetectors could be implemented within a broad wavelength range with the aid of appropriate LCs. Figure 1a illustrates the schematic device architecture of the LC-integrated heterostructure photodetector. It resembles a miniaturized conventional solar concentrator, for there is no need to collect the incident light over a large area. The PQDs are embedded in a transparent PMMA waveguide matrix, which can avoid re-absorption of the propagating luminescence. The reflectors help to recycle the escaped photons back into the waveguide, thereby reducing the escape cone losses. Upon illumination, the incident light will refract into polymer to shed on the PQD luminophores. Due to the 5*d* high energy states of Ce^3+^ ions, the PQDs have strong UV absorption in the region of 200–300 nm. Then, the efficient energy transfer process is triggered to convert the UV light to visible luminescence with high photoluminescence quantum yields (PLQY) as presented in Fig. 1b, subsequently reabsorbed by the integrated photodetector. Since the emitted luminescence can be tuned to match the peak response of the photodetector, the resultant quantum yield and detectivity in the deep UV region would be boosted substantially, achieving the same level as the Vis-NIR lights.

**Fig. 1:**
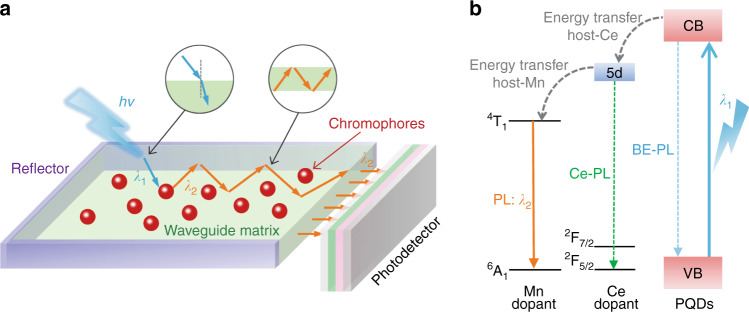
Working mechanism of the luminescent concentrator for photodetection. **a** Schematic device architecture of the LC-coupled heterostructure photodetector. The insets above indicate the incident light refraction at PMMA surface and waveguiding of emitted light by total internal reflection, respectively. **b** Energy transfer process of Cr^3+^, Ce^3+^, Mn^2+^ doped CsPbCl_3_ PQDs. The band edge, Ce^3+^ and Mn^2+^ PL followed by excitation (*λ*_1_) are shown by the blue dash, green dash and orange solid arrows, respectively. High PLQY Mn-PL (*λ*_2_) is due to the efficient energy transfer from PQDs.

The strategy of attaining efficient broadband photodetection in this work is the synthesis of suitable chromophores and waveguide matrices, which is of great importance. On the one hand, the smooth surface of the concentrator can suppress the scattering loss and concentrate the incident diffuse light. The frequently used waveguide materials PMMA may also be substituted with fluorinated polymers, the C–H overtone absorption would then be further refrained with minimal photon loss^10^. On the other hand, it is pivotal to engineer the sufficiently large Stokes shift, which is the difference between the optical absorption edge and the emission band of chromophores, through various materials design strategies, thereby minimizing the self-absorption loss and achieving a high PLQY^11^. This scheme would be well accomplished by adopting the PQDs with multi-dopants, as demonstrated above. The energy funneling provided by the multiple quantum wells in PQDs will produce a wide Stokes shift and high PL^12^.

The technical principle in this work can inspire us to design highly efficient photodetectors more wisely and flexibly within a broader wavelength range in the future. For example, by employing the up-conversion nanoparticles as chromophores, the spectral response range of photodetectors can be potentially expanded to mid-Infrared or even far-Infrared. Moreover, with the discovery and exploration of nanocrystal scintillators, incorporation of these scintillators in LCs-coupled photodetectors can realize radiation detection as well. This technique provides a promising solution for developing highly efficient and ultra-broadband optoelectronics by continually engineering the luminophores and concentrator architectures.
